# The effect of a blue enriched white light on salivary antioxidant capacity and melatonin among night shift workers: a field study

**DOI:** 10.1186/s40557-018-0275-3

**Published:** 2018-10-19

**Authors:** Reza Kazemi, Rasoul Hemmatjo, Mokarami Hamidreza

**Affiliations:** 1 Research Center for Health Sciences, Institute of Health, Shiraz University of Medical Sciences, Shiraz, Iran; 2 Department of Ergonomics, School of Health, Shiraz University of Medical Sciences, Razi avenue, Shiraz, Iran; 3 Department of Occupational Health Engineering, School of Health, Urmia University of Medical Sciences, Urmia, Iran

**Keywords:** Blue enriched white light, Salivary antioxidant night shift, Melatonin

## Abstract

**Background:**

Because of their positive impact on individuals’ performance and alertness, blue enriched white light sources are gaining popularity in households and industries. However, these sources of blue light spectrum may cause oxidative stress. On the other hand, there are no empirical studies investigating the negative effect of blue enriched white light on oxidative stress. Thus, the current study aimed at empirical assessment of the effect of such light sources on oxidative stress among night shift workers.

**Methods:**

The study, which adopted a cross-sectional design, focused on 30 control room operators of a petrochemical complex. The subjects followed a shift-work schedule comprising 7 night shifts, 7 day shifts, and 7 days off. The subjects were exposed to 6500 K, 3000 K, and 17,000 K light sources (which have various degrees of blue light) during three consecutive work cycles, with each cycle lasting for 7 nights. In each light condition, three salivary measurements were conducted (at the beginning, in the middle, and at the end of the shift). The measurements were used to assess catalase (CAT), total thiol molecules (TTG), and total antioxidant capacity (TAC), and melatonin.

**Results:**

The results of repeated measures ANOVA showed that there was no significant difference among various light conditions with regard to salivary biomarkers (catalase, total thiol molecules, and total antioxidant capacity). There was however a significant difference between 3000 K and 17,000 K conditions with regard to the concentration of salivary melatonin (*p* = 0.001).

**Conclusion:**

Given that there was no significant difference among various light conditions in terms of biomarkers, it is concluded that using sources of light with high color temperature can be recommended. Nonetheless, because of the limitations of the present study (e.g. short period of intervention), it is suggested that care should be exercised in using such light sources.

## Background

Visible light, which constitutes a small proportion of electromagnetic spectrum, is detectable by human eyes [1]. Blue light, which has a wavelength of 380–495 nm, belongs to the spectrum of the shortest, highest energy wavelengths in the visible light spectrum. This range of light wavelength is known as high-energy visible (HEV) light in the visible spectrum range [2]. HEV (which entails the blue light) is the shortest wavelength that can reach the retina of the oculus [3].

Nowadays, light-generating devices that emit short-wave energy (e.g. fluorescent and compact fluorescent) are common [4, 5]. Research has revealed that exposure to blue-enriched white light can promote workers’ alertness, performance, and mood [6]. It can also enhance sleep quality and perceived functioning outside the work place [7]. It has also been demonstrated that exposure to blue-enriched white light during night shift works declines sleepiness and salivary melatonin to a great extent and causes improvement in cognitive performance [8].

On the other hand, the great stress that this type of light imposes on oculus and body to reach the retina is regarded as a downside [9–13]. Indeed, the mechanism of this stress entails reactive oxygen species (ROS) produced by excited blue light in the retina photoreceptors [14–16]. This mechanism is known as oxidative stress, which is defined as the imbalance between ROS production and antioxidant defense inside human organism. Oxidative stress may lead to retinal diseases [17]. It also plays a crucial pathogenetic role for non-communicable diseases. More specifically, oxidative stress destroys lipids and DNA and inhibits/deactivates proteins with a consequent disruption of overall biological function [18].

Research findings indicated that the irradiation of mammalian cells with blue light leads to hydrogen peroxide (H2O2) production and DNA damage [19]. Additionally, blue light irradiation is blamed for apoptosis or mitochondrial dysfunction in mammalian fibroblasts and reduction in the viability of corneal epithelial cells. Further, irradiation at 410 and 480 nm leads to reactive oxygen species production [18].

Thus, the available research evidence provides support for the harmful impacts of blue spectrum of visible light (e.g. oxidative stress and eye-related problems). Nonetheless, all these studies have adopted an in vivo, in vitro, or experimental design focusing on pure blue light. It is unclear whether a blue enriched white light emitted in the actual workplace can cause oxidative stress. To address this gap in the literature, the current study aimed at assessing oxidative stress caused by the irradiation of blue enriched white light in the workplace.

## Methods

### Participants

The study was carried out among 30 subjects working as petrochemical control room operators located in Iran. They were all males, with a mean age of 30.2 years (SD = 4.1) and a mean night-shift work experience of 4.5 years (SD = 1.8). All the participants met the following criteria: no one suffered from any diabetes, cardiovascular disease, high blood pressure and excessive exercise; none of them was a smoker. All procedures for this investigation were approved by the Ethics Committee and Vice Chancellor of Research of Shiraz University of Medical Sciences.

### Study design and procedure

A field trail interventional and within-subjects design was adopted in the current study. There were three light treatments (baseline, 6500 K, and 17,000 K) and the research was completed in three stages between 21 January and 20 March, 2015 (hence, the entire study was completed in 9 weeks). The studied rooms were identical considering the intensity of light exposure and the responsibilities defined for the staff members. The subjects’ shift-work schedule comprised a 21-day cycle (7 night shifts, 7 day shifts, and 7 days off) and every shift lasted for 12 h. In addition, in order to eliminate the intervening effect of adaptation to doing several night shifts in a row, in all the three stages, assessments were carried out during the seventh consecutive night shift. In other words, in each stage of assessment, participants had already been exposed to the target type of light for seven consecutive nights. In total, the study was conducted in three cycles, encompassing 9 weeks in a row.

Additionally, in order to control the influence of circadian stimuli, the participants were advised not to drink caffeinated drinks from 4 h prior to starting their shift work until the end of the shift. Furthermore, care was taken to exclude alcoholic staff members from the study. The participating workers were also requested to avoid taking naps before and during the study and to have regular sleep schedules during off days. It should be noted that, since staff members were living in camps constructed by the company far from their family, they all followed a relatively similar sleep-wake schedule from 8 a.m. to 3 p.m.

To assess the non-visual impacts of blue-enriched white light, two different fluorescent light sources (17,000 K and 6500 K) were applied. The light sources consisted of fluorescent tubes with high color temperature (17,000 K Philips, ActiViva Active, TLD 36 W), which is called cold light, and medium color temperature (6500 K Philips, 36 W), which is known as day light. To create identical conditions (in terms of the available light sources), 36 W intervention light sources were used. Light sources were changed prior to the beginning of each work cycle. Baseline measurements were conducted under the available lighting conditions (2500–3000 K) in the seventh night shift. Subsequently, the light sources were replaced by 6500 K fluorescent lamps at the beginning of the second work cycle. Second-stage assessments were carried out at the seventh night shift. Finally, at the beginning of the third work cycle, 17,000 K light sources were installed and the third round of measurements was conducted at the seventh night of the cycle.

### Method

At the end of the 7th night shift (6–7 a.m.) in all three stages, the salivary samples were collected in an unstimulated way from each subject and were transferred into sterile tubes. Frozen salivary samples were thawed and analyzed on ice immediately, then centrifuged at 3000 RPM. The supernatant was used for analysis of catalase (CAT), total thiol molecules (TTG), and total antioxidant capacity (TAC).

### Assay of total antioxidant capacity

FRAP test was used to assess antioxidant capacity. In this test, the amount of Fe3+ to Fe2+ reduction is measured. That is, the medium is exposed to Fe3+ and the antioxidants which are available in the medium begin to produce Fe2+ as an antioxidant activity. The reagent containing TPTZ is dissolved in acetate buffer (pH 3.6) and FeC13. The complex between Fe2+ and TPTZ generates a blue color with absorbance at 593 nm, which is assessed based on a calibration curve obtained by different concentrations of FeC13 [20].

### Assay of CAT

Catalase activity was estimated via spectrophotometric ally on saliva and expressed in units per milliliter. CAT activity was gauged in samples by assessing the absorbance decline at 240 nm in a reaction medium containing 1682 10 nM H2O2, and 50 mM sodium phosphate buffer (pH 7.0). One unit of the enzyme consists of 1 M H2O2 consumed/min, with the specific activity being reported as units/ml saliva [21].

### Assay of Total thiol molecules (TTG)

Salivary protein thiol was measured via spectrophotometric method using dithionitrobenzene (DTNB)-Ellman’s method [15]. Ellman’s reagent or 5,5′-dithiobis (2-nitrobenzoate, DTNB), which is a symmetrical aryl disulfide, undergoes the thiol-disulfide interchange reaction when a free thiol is present [16]. In comparison with both disulfides, the TNB dianion has a rather intense absorbance at 412 nm. The protein thiol concentration in saliva was assessed using the molar extinction coefficient of the TNB complex in the assay mixture at 412 nm, which is obtained upon using known standard concentrations and their absorbance values [22].

### Assay of salivary melatonin

In this study, salivary melatonin (which contains approximately 30% of plasma melatonin) was used to determine the level of melatonin. Because of its non-invasive nature, nowadays a larger number of researchers are using this method for measuring melatonin [23]. The samples were collected at four times during the night shift (7 p.m., 11 p.m., 3 a.m., and 7 a.m.) via a saliva sample collector (Sartsert, Germany). With the aim of minimizing the intervening effect of food consumption on the melatonin level, participants were asked not to eat anything for at least 1 h before collecting samples. The collected samples were immediately centrifuged, frozen, and stored at − 20 °C and were subsequently transferred to the laboratory. ELIS kit (manufactured by Biotech Company in China) was used to measure melatonin levels. The sensitivity of the tests was 1.6 ± 1.3 pg/ml. Intra-assay coefficient of variation was 8.1% at 1.8 pg/ml and 5.5% at 25 pg/ml. In all the three stages of the research (before and after the intervention), participants’ salivary melatonin was assessed 3 times (in 6 h intervals) during the shift.

### Statically analysis

The collected data were analyzed by Statistical Package for the Social Sciences (SPSS) 21 (SPSS Inc., Chicago, IL, USA). The Kolmogorov-Smirnov test was used to assess the normality of data distribution. The effect of lighting source on all measurements was tested by a repeated measures analysis of variance (ANOVA) for each of the dependent variables to determine if there were any significant differences between three phases. The statistical significance was set at 0.05.

## Results

Table 1 shows the characteristics of the light sources used in this study. As observed, we used three light sources with different color temperatures, which were almost identical in intensity. As well as Table 2 shows characteristics and baseline variables of the subjects.

**Table 1:** Characteristics of the used light sources

Light source	Correlated colour temperature (K)	Illuminance on the work plane (in lx)^a^	Chromaticity coordinates 1931 CIE 28 (x, y)
Fluorescent (18 W)	3000 k	510	0.453, 0.397
Fluorescent (18 W)	6500 k	501	0.447, 0.422
Fluorescent (18 W)	17,000 k	506	0.450, 0.440

^a^Mean illuminance levels

**Table 2:** characteristics and baseline variables of the subjects

Variables	m (SD)
age	31.10 ± 2.60
Shift work experience (year)	6.30 ± 1.00
Body mass index (BMI)	24.2 (3.8)
Sleep quantity (h)	7.00 ± 1.12
Sleep quantity	6.70 ± 3.62
Light (lx)	530.50 ± 34.20

**Fig. 1:**
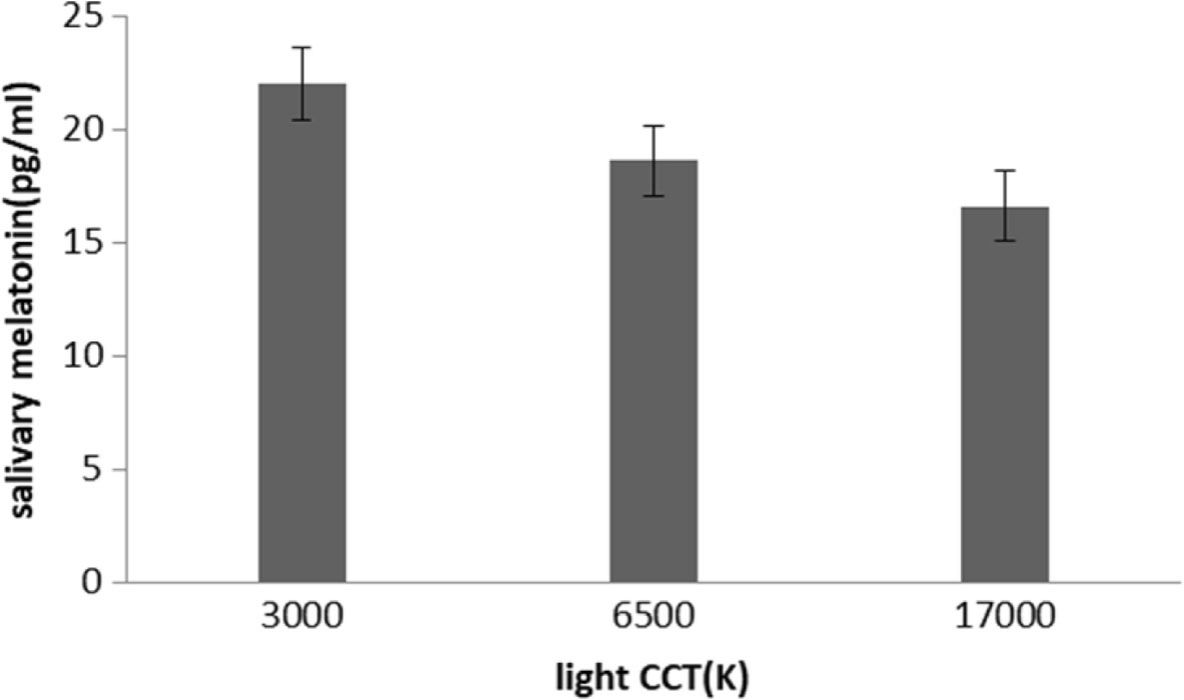
Effects of light on salivary melatonin (mean and SE)

Figure 1 and Table 3 illustrates the influence of light condition on the salivary melatonin. In our analysis, significant differences were found between these three environments (CCT of light) [F (1.5,45) = 6 *P* = 0.00 9]. Further analysis showed that the salivary melatonin concentration under 17,000 k was significantly lower than that under 3000 k (*p* < 0.001).

**Table 3:** Results from an analysis of covariance for repeated measures

variable	Light color temperature			F	p
	3000 (k)	6500 (k)	17,000 (k)		
FRAP	592 (150)	6117 (114)	595 (164)	0.2	0.6
protein thiols	300 (91)	250 (87)	257 (97)	1.2	0.3
catalase activity	2.4 (1.2)	2.7 (1.1)	2.4 (1.3)	0.7	0.5
Salivary melatonin	22 (6)	18.6 (5.5)	16.5 (7)	6	0.009

**Fig. 2:**
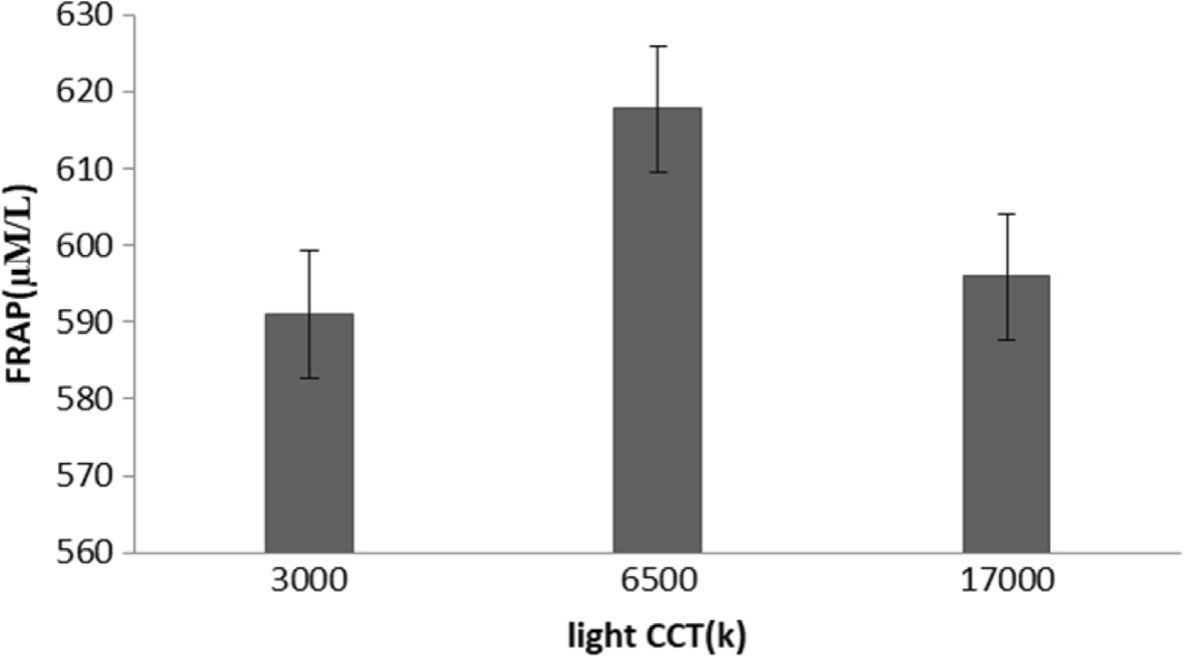
Effects of light on TAC (mean and SE

The results of repeated-measures ANOVA also showed that the FRAPs among the three environments was not significantly different [F (2,29) = 0.2 *P* = 0.6] (Fig. 2 and Table 3).

**Fig. 3:**
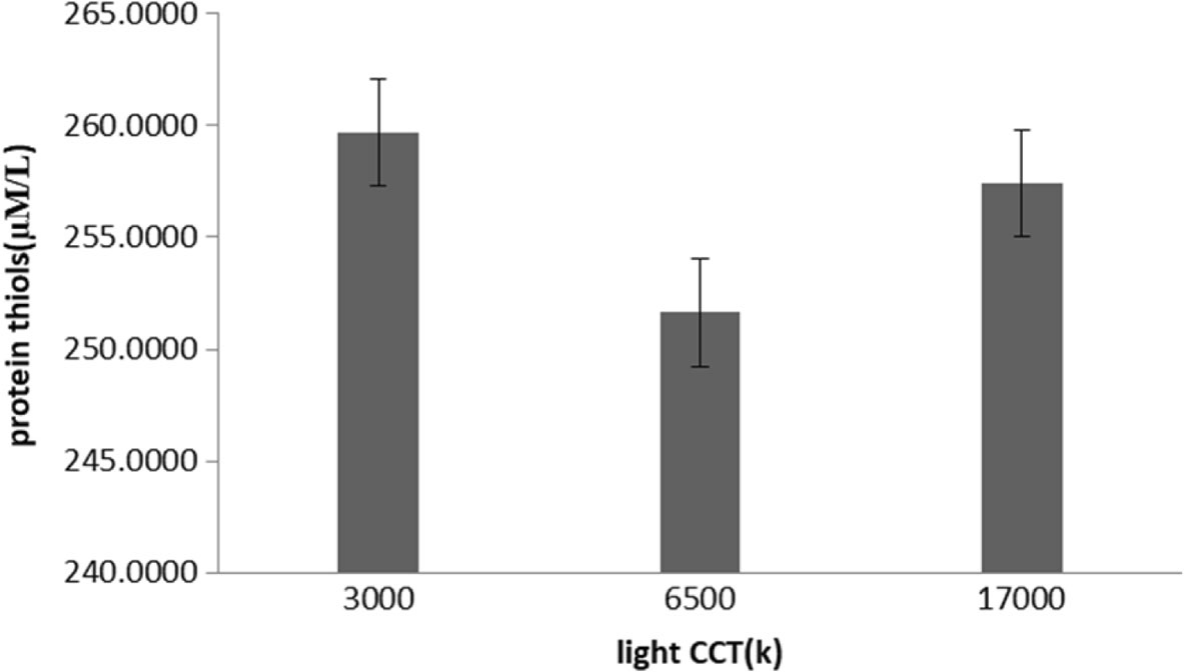
Effects of light on protein thiols (mean and SE)

**Fig. 4:**
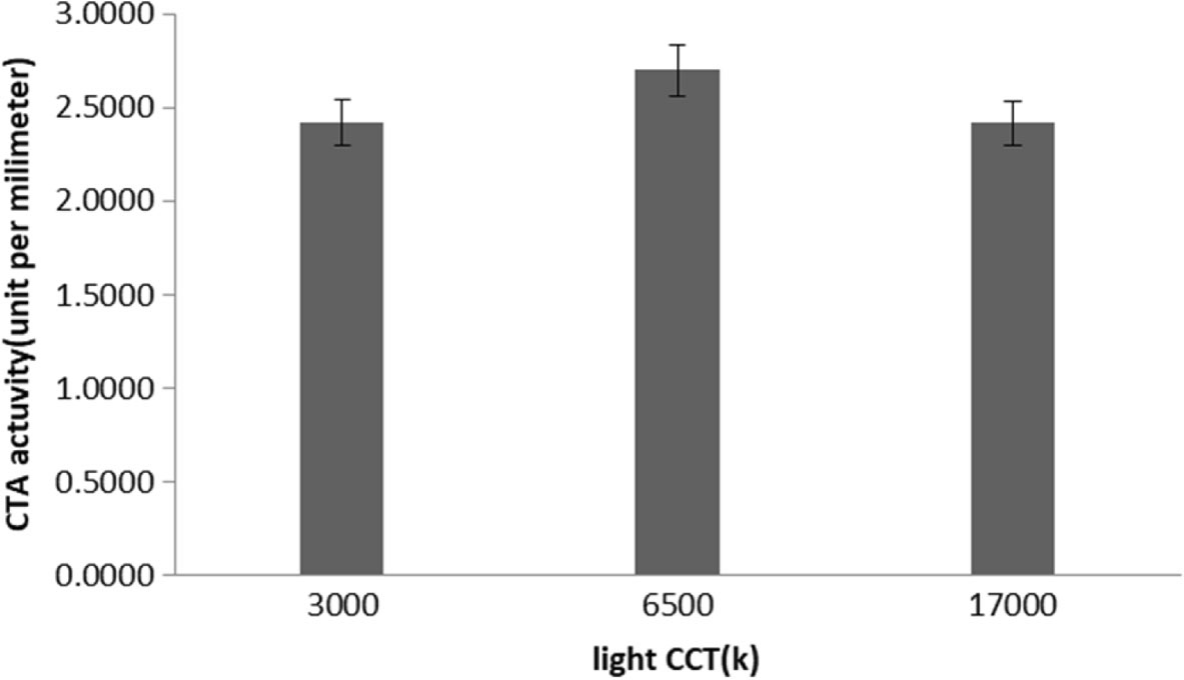
Effects of light on CTA activity (mean and SE)

Similarly, statistical analysis yielded no significant differences between protein thiols (Fig. 3 and Table 3) [F (2, 92) = 1.2; *p* = 0.3] and catalase activity [F (2,58) = 0.7 *P* = 0.5] among the three environments (CCT of light) (Fig. 4 and Table 3).

## Discussion

One of the concerns about the light sources is their impact on body antioxidants through suppressing melatonin, which is the most powerful antioxidant inside the body [8]. Research has revealed that visible light with short wavelength plays a significant role in melatonin suppression and oxidative stress [24]. New light sources are aimed at generating light with high color temperature to improve lighting [6]. It is therefore crucial to empirically investigate the effect of light color temperature on oxidative stress given that higher color temperatures increase the percentage of blue light. Thus, the present study, which is one of the fewest ones in this area, aimed at assessing the impact of light sources with various color temperatures on oxidative stress among night shift workers.

The most important strength of the current study was its empirical nature, a research design that has not been previously followed. In the present study, total thiol molecules (TTG) and total antioxidant, catalase (CAT), and capacity (TAC) were used as indicators of oxidative stress under various light conditions (in terms of the temperature of light color).

The results showed that blue color has no influence on the capacity of total thiol molecules (TTG), catalase (CAT), and capacity (TAC) of the saliva. That is, increasing light color temperature, which leads to the enhancement of light intensity, does not have any significant effect on the antioxidative capacity. Although no study was found with a completely similar design (i.e. empirically investigating the impact of light with various color temperatures on the antioxidative capacity), the results of this study are in conflict the findings of related research projects. Jun-Hai Yang et al., for example, probed into the photoreceptor ellipsoids generated by reactive oxygen species (ROx) after blue light illumination displayed that blue light-induced generation of reactive oxygen species in photoreceptor ellipsoids demands mitochondrial electron transport [9].

In another study, Fumihiko Yoshino et al. investigated the impact of dental resin curing blue light on oxidative stress. They discovered that blue light irradiation enhanced the level of lipid peroxidation (measured by malondialdehyde) in isolated rat aorta blood vessels. Also, cell proliferative activity declined in the course of time and apoptosis of human aorta vascular smooth muscle cells (VSMCs) was induced. The results revealed that ROS (like hydrogen peroxide and hydroxyl radicals) were produced in VSMCs through blue light irradiation. They in turn induced cytotoxicity connected with oxidative stress, which increased lipid peroxidation and apoptosis [25].

In addition, Ayaka Yoshida et al. demonstrated that ROS generation in rat gingival tissue induced blue light irradiation oxidative stress. The researchers suggested that, through inducing oxidative stress and consuming a significant amount of intracellular glutathione, blue light irradiation at clinical levels of tooth bleaching treatment can increase lipid peroxidation [26].

Several justifications can be presented to explain the contradiction between the findings of the present study and those of other research projects. First, previous studies focused on investigating the effect of pure blue light with high intensity (400–480 nm) [9, 26]. Conversely, in the current study, the effect of blue enriched white light was assessed. Indeed, the visible light spectrum of light sources in this study was complete (400–780 nm) and the proportion of blue light was enhanced only through the color temperature of light sources. In contrast, pure light spectrum with high intensity was used in previous studies [9, 25, 26].

Another reason for the contradiction between the findings of this study and the previous ones’ is that past researches investigated the in vivo or in vitro effect of blue light on oxidative stress [9, 25, 26]. The current study, however, empirically investigated this effect among humans in a real workplace. In vitro research does not provide the opportunity to produce antioxidants in reaction to oxidative stress, while human body is capable of adaptation to internal changes during an empirical study. In real settings, internal body adaptors (e.g. melatonin) and even external materials containing antioxidants are able to deal with blue light induced oxidants [27].

Finally, the current study concentrated on assessing the capacity of available antioxidants. In contrast, previous studies mainly focused on the effects of oxidative stress. They also used plasma samples and other body tissues to measure the capacity of antioxidants, a more reliable criterion than salivary sample [9, 15]. Since the present study has an empirical nature, it cannot be readily compared with other researches. Of course, the major superiority of this study (compared with other ones) is that it is the only piece of empirical research investigating the influence of blue light on the capacity of body antioxidant. The results can form a basis for further studies.

### Limitations of the study

One of the limitations of the study is the short intervention period (1 week for each light condition). Due to ethical issues, the researchers did not have the permission for longer interventions. It is therefore suggested that future researchers try to examine the effect of longer interventions.

Another limitation of the study was that the researchers could not control the amount of nutritive intake that contained antioxidants. Perhaps using such foods moderated oxidative stress.

Assessing oxidative stress via corneal sampling is certainly more useful because eye tissues are strongly influenced by light. However, because collecting samples from the corneal is an invasive procedure, the researchers were only confined to salivary oxidative stress.

## Conclusion

The results of this study show that blue light has no effect on oxidative stress. Therefore, since blue light has positive impact on night shift workers’ alertness and performance, it can be used as a suitable and safe performance enhancement solution. Nevertheless, since the body’s antioxidant capacity may be influenced by blue light, further studies with longer interventions are required to make firmer claims.

## References

[1] Deng W, Goldys EM (2012). Plasmonic approach to enhanced fluorescence for applications in biotechnology and the life sciences. Langmuir.

[2] Grimm C, Wenzel A, Williams TP, Rol PO, Hafezi F, Remé CE (2001). Rhodopsin-mediated blue-light damage to the rat retina: effect of photoreversal of bleaching. Invest Ophthalmol Vis Sci.

[3] Krinsky NI (2002). Possible biologic mechanisms for a protective role of xanthophylls. J Nutr.

[4] Chellappa SL, Steiner R, Blattner P, Oelhafen P, Götz T, Cajochen C (2011). Non-visual effects of light on melatonin, alertness and cognitive performance: can blue-enriched light keep us alert?. PLoS One.

[5] Paul MA, Miller JC, Gray G, Buick F, Blazeski S, Arendt J (2007). Circadian phase delay induced by phototherapeutic devices. Aviat Space Environ Med.

[6] Mills PR, Tomkins SC, Schlangen LJ (2007). The effect of high correlated colour temperature office lighting on employee wellbeing and work performance. J Circadian Rhythms.

[7] Viola AU, James LM, Schlangen LJ, Dijk D-J (2008). Blue-enriched white light in the workplace improves self-reported alertness, performance and sleep quality. Scand J Work Environ Health.

[8] Motamedzadeh M, Golmohammadi R, Kazemi R, Heidarimoghadam R (2017). The effect of blue-enriched white light on cognitive performances and sleepiness of night-shift workers: a field study. Physiol Behav.

[9] Yang J-H, Basinger SF, Gross RL, Wu SM (2003). Blue light–induced generation of reactive oxygen species in photoreceptor ellipsoids requires mitochondrial electron transport. Invest Ophthalmol Vis Sci.

[10] Lee J-B, Kim S-H, Lee S-C, Kim H-G, Ahn H-G, Li Z, Yoon KC (2014). Blue light–induced oxidative stress in human corneal epithelial cells: protective effects of ethanol extracts of various medicinal plant MixturesBlue light-induced oxidative stress in the cornea. Invest Ophthalmol Vis Sci.

[11] Margrain TH, Boulton M, Marshall J, Sliney DH (2004). Do blue light filters confer protection against age-related macular degeneration?. Prog Retin Eye Res.

[12] Sparrow JR, Cai B (2001). Blue light–induced apoptosis of A2E-containing RPE: involvement of caspase-3 and protection by Bcl-2. Invest Ophthalmol Vis Sci.

[13] Taylor HR, West S, Muñoz B, Rosenthal FS, Bressler SB, Bressler NM (1992). The long-term effects of visible light on the eye. Arch Ophthalmol.

[14] Beatty S, Koh H-H, Phil M, Henson D, Boulton M (2000). The role of oxidative stress in the pathogenesis of age-related macular degeneration. Surv Ophthalmol.

[15] Winkler BS, Boulton ME, Gottsch JD, Sternberg P (1999). Oxidative damage and age-related macular degeneration. Mol Vis.

[16] Cai J, Nelson KC, Wu M, Sternberg P, Jones DP (2000). Oxidative damage and protection of the RPE. Prog Retin Eye Res.

[17] Wagner K-H, Brath H (2012). A global view on the development of non communicable diseases. Prev Med.

[18] Godley BF, Shamsi FA, Liang F-Q, Jarrett SG, Davies S, Boulton M (2005). Blue light induces mitochondrial DNA damage and free radical production in epithelial cells. J Biol Chem.

[19] Hockberger PE, Skimina TA, Centonze VE, Lavin C, Chu S, Dadras S, Reddy JK, White JG (1999). Activation of flavin-containing oxidases underlies light-induced production of H2O2 in mammalian cells. Proc Natl Acad Sci.

[20] Benzie IF, Strain JJ (1996). The ferric reducing ability of plasma (FRAP) as a measure of “antioxidant power”: the FRAP assay. Anal Biochem.

[21] Sinha AK (1972). Colorimetric assay of catalase. Anal Biochem.

[22] Hu M-L (1994). [41] Measurement of protein thiol groups and glutathione in plasma. Methods Enzymol.

[23] Mirick DK, Davis S (2008). Melatonin as a biomarker of circadian dysregulation. Cancer Epidemiol Prev Biomarkers.

[24] Rea MS, Bullough JD, Figueiro MG (2002). Phototransduction for human melatonin suppression. J Pineal Res.

[25] Yoshino F, Yoshida A, Okada E, Okada Y, Maehata Y, Miyamoto C, Kishimoto S, Otsuka T, Nishimura T, MC-i L (2012). Dental resin curing blue light induced oxidative stress with reactive oxygen species production. J Photochem Photobiol B Biol.

[26] Yoshida A, Shiotsu-Ogura Y, Wada-Takahashi S, Takahashi S-S, Toyama T, Yoshino F (2015). Blue light irradiation-induced oxidative stress in vivo via ROS generation in rat gingival tissue. J Photochem Photobiol B Biol.

[27] Wilking M, Ndiaye M, Mukhtar H, Ahmad N (2013). Circadian rhythm connections to oxidative stress: implications for human health. Antioxid Redox Signal.

