# Correction to: The effect of a blue enriched white light on salivary antioxidant capacity and melatonin among night shift workers: a field study

**DOI:** 10.1186/s40557-018-0280-6

**Published:** 2018-12-13

**Authors:** Reza Kazemi, Rasoul Hemmatjo, Hamidreza Mokarami

**Affiliations:** 1 Research Center for Health Sciences, Institute of Health, Shiraz University of Medical Sciences, Shiraz, Iran; 2 Department of Ergonomics, School of Health, Shiraz University of Medical Sciences, Razi avenue, Shiraz, Iran; 3 Department of Occupational Health Engineering, School of Health, Urmia University of Medical Sciences, Urmia, Iran

**Correction to : Ann Occup Environ Med**

**https://doi.org/10.1186/s40557-018-0275-3**

In the original publication of this article [1], the corresponding author’s name ‘Mokarami Hamidreza’ should be changed to ‘Hamidreza Mokarami’.

The publisher apologies for this error. The original publication has been corrected.
